# Comparison of quantitative polymerase chain reaction, Kato-Katz and circulating cathodic antigen rapid test for the diagnosis of *Schistosoma mansoni* infection: A cross-sectional study in Kirinyaga County, Kenya

**DOI:** 10.1016/j.crpvbd.2021.100029

**Published:** 2021-07-15

**Authors:** Benard Chieng, Collins Okoyo, Elses Simiyu, Paul Gichuki, Cassian Mwatele, Stella Kepha, Sammy Njenga, David Mburu

**Affiliations:** a Eastern & Southern Africa Centre of International Parasite Control (ESACIPAC), Kenya Medical Research Institute (KEMRI), PO BOX 54840-00200, Nairobi, Kenya; b Department of Microbiology, Biotechnology and Biochemistry, Kenyatta University, Nairobi, Kenya

**Keywords:** *Schistosoma mansoni*, Kenya, Comparative performance study, Kato-Katz, POC-CCA, qPCR

## Abstract

The current standard diagnostic tests for *Schistosoma mansoni* are the Kato-Katz and circulating cathodic antigen (CCA) techniques. However, these techniques have been documented to have several limitations that have a direct impact on schistosomiasis control programmes. Therefore, there is a need for more sensitive and specific tests for diagnosing schistosomiasis. This study compared the performance of quantitative polymerase chain reaction (qPCR), Kato-Katz, and point-of-care circulating cathodic antigen (POC-CCA) techniques in the diagnosis of *S. mansoni* infection in the Mwea irrigation scheme, Kirinyaga County in Central Kenya. We carried out a cross-sectional study on 357 individuals residing in four villages in the Mwea irrigation scheme. The participants provided urine and stool samples which were screened for *S. mansoni* infections using the three techniques. The prevalence of *S. mansoni* by each technique was calculated and 95% confidence intervals estimated using binomial regression model. Sensitivity and specificity were determined using 2 × 2 contingency tables and compared using the McNemar’s chi-square test. Positive and negative predictive values were also determined using the weighted generalized score chi-square test for paired data. The study showed that the prevalence of *S. mansoni* was 32.8%, 62.5% and 72.8% using Kato-Katz, POC-CCA and qPCR techniques, respectively. Further, when using Kato-Katz as a gold standard, POC-CCA sensitivity was 78.6% and specificity was 45.4%, while qPCR sensitivity was 97.4% and specificity was 39.2%. When using qPCR as the gold standard, Kato-Katz sensitivity was 43.8% and specificity was 96.9%, while POC-CCA sensitivity was 78.1% and specificity was 79.4%. Finally, when using the averaged results from the three techniques as the gold standard, the sensitivity was 41.6%, 79.4% and 92.5% for Kato-Katz, POC-CCA and qPCR, respectively, with a specificity of 100% for all techniques. Kato-Katz technique showed low sensitivity compared to the POC-CCA and qPCR despite it being the most commonly preferred method of choice to diagnose *S. mansoni* infections. qPCR showed superior sensitivity followed by POC-CCA, hence it can be used as an alternative or to confirm the results obtained by the Kato-Katz technique.

## Highlights

• Comparison of the performance of Kato-Katz, POC-CCA and qPCR for diagnosis of *S. mansoni* in Kirinyaga County, Kenya.
• A total of 357 urine and stool samples were tested for *S. mansoni* infection.
• qPCR estimated the highest prevalence followed by POC-CCA and Kato-Katz.
• qPCR showed a generally high sensitivity and specificity.

## Graphical abstract

**Figure:**
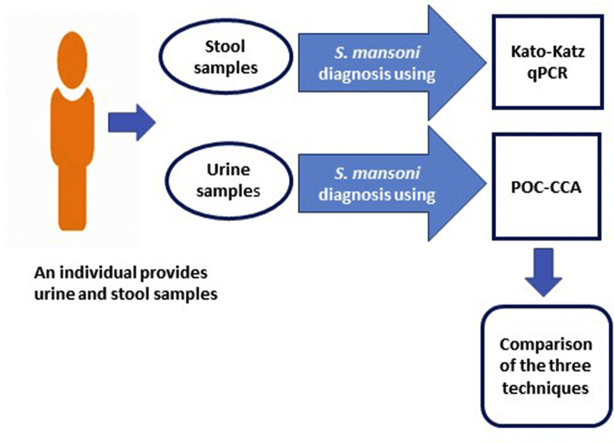

## 1 Introduction

Schistosomiasis caused by *Schistosoma* parasites is classified among neglected tropical diseases (NTDs) (Steinmann et al., 2006; Clark et al., 2019). About 200 million people are infected with schistosomes globally, while about 800 million people are at risk of infection in 74 countries (Steinmann et al., 2006). The disease predominantly affects children, farmers and women who come into contact with waterbodies which may harbour the infected intermediate host snails (Kildemoes et al., 2017). Transmission occurs when individuals with schistosomiasis contaminate water sources with their feces or urine containing eggs of *Schistosoma* spp. (Hotez et al., 2014; WHO, 2020). Schistosomiasis is prevalent in subtropical and tropical regions, especially in communities where people live without access to adequate sanitation and safe drinking water (WHO, 2020). Chronic schistosomiasis, especially the intestinal schistosomiasis, can result in impaired growth development outcomes in children and can lead to death, making it one of the most significant neglected tropical diseases. The World Health Organization (WHO) advocates for the control of schistosomiasis in endemic areas by regular mass drug administration (MDA) to the most at-risk groups. According to a recent report by the WHO, about 229 million individuals required preventive chemotherapies in 2018 worldwide (WHO, 2020).

The most common laboratory methods used for schistosomiasis detection are based on standard microscopy, i.e. visual observation of *Schistosoma* eggs from stool and urine, Kato-Katz technique for *Schistosoma mansoni*, and urine filtration technique for *Schistosoma haematobium*. The Kato-Katz technique is relatively inexpensive, easy to perform, and is the method recommended by the WHO and the method of choice in regions with moderate to high infection intensity due to its ability to provide both intensity and prevalence data (WHO, 2011). However, in regions with low endemicity (i.e. low prevalence and intensity of infection), the Kato-Katz method is less accurate due to day-to-day and intra-stool variations (Kittur et al., 2016; Taman & El-Beshbishi, 2019). Therefore, its use may lead to underestimation of disease prevalence after MDA.

WHO recommends that MDA programmes should give priority to preschool-aged children (PSAC), school-aged children (SAC) and women of reproductive ages (WRA) (WHO, 2012). Recent WHO data have shown that about 120.1 million PSAC, 456.3 million SAC and 127.9 million WRA have received treatment for soil-transmitted helminths (STH) in 2018 worldwide (Deol et al., 2019). The Kenyan government through the Ministry of Health and the Ministry of Education started in 2012 a school-based deworming programme targeting children residing in STH- and schistosome-endemic areas (Brooker et al., 2010). The implementation of this programme was expected to reduce the disease prevalence, although incidence of reinfection has been documented (Okoyo et al., 2016). The observed reinfection can be attributed to the exclusion of the other groups and/or the use of a less sensitive diagnostic technique (Truscott et al., 2014).

There have been several methods developed and tried in the diagnosis of *S. mansoni*, including circumoval precipitin test on serum samples (Noya et al., 2002; Carvalho Do Espiŕito-Santo et al., 2014), the FLOTAC technique on stool samples (Glinz et al., 2010), and POC-CCA for the detection of *Schistosoma* antigen in urine samples (Sousa-Figueiredo et al., 2009; Ochodo et al., 2015; Utzinger et al., 2015). The POC-CCA test depends on the detection of major antigens released by viable worms. The method is easy to use in the field and needs minimal practical training. The challenges of using POC-CCA test are that it is prone to giving false positive results (Ashton et al., 2011), estimates inaccurately disease prevalence (Chernet et al., 2017) in light *S. mansoni* infections, has a high level of cross-reactivity, the positivity of the test is strongly correlated with *Schistosoma* infection intensity, and the sensitivity also decreases in low infection settings (Tchuem Tchuenté, 2011).

Considering the limitations of the above-mentioned techniques, a more sensitive and specific diagnostic method should be considered for the detection of *S. mansoni*. Polymerase chain reaction (PCR) method has been used for the detection of a wide range of different parasites (Haggag and Abdullah, 2011). This technique is based on the detection of *Schistosoma* spp. deoxyribonucleic acid (DNA) in various samples such as serum, plasma, stool and urine samples by amplifying 121-bp tandem repeat (Haggag & Abdullah 2011; Song et al., 2015). It involves DNA extraction from eggs before the amplification process. The present study compared quantitative PCR, Kato-Katz and point-of-care circulating cathodic antigen performance in detection of *S. mansoni* infections.

## 2 Materials and methods

### 2.1 Study area

The study was conducted in the Mwea Irrigation Scheme located in Kirinyaga County, approximately 100 km north-east of Nairobi, Kenya (KNBS, 2010). The study area is the country’s largest irrigation scheme established in 1954 and falls within two sub-counties: Mwea West and Mwea East (KNBS, 2010). Mwea West sub-county, the study’s focus, has two locations, Thiba and Kangai, located along the drainage basin of two rivers, River Thiba and River Nyamindi, and is endemic for both STH and *S. mansoni.* The villages included in the study were purposively sampled based on *S. mansoni* endemicity reported in a previous study (Masaku et al., 2015) and in consultation with the Public Health Officer of the area. Four villages were included in the study: Gakungu, Kasarani, Kiratina and Rurumi.

### 2.2 Study design and sample size

A cross-sectional study was conducted between August and September 2019. The study used a 55% schistosomiasis prevalence based on a previous study (Masaku et al., 2015), a level of significance and an error margin of 0.05, and standard deviation at 95% CI (1.96) to calculate a minimum sample size of 380 individuals by using the formula by Fisher (1934). Each selected participant was asked to provide a stool and urine sample.

### 2.3 Study population and selection criteria

Thiba location in Mwea West sub-county was purposively selected to participate in the study as there is a previous related study in the area (KEMRI/SERU/3326) by Gichuki et al. (2019). The location has a total of eight villages: Mbui Njeru, Maendeleo, Gakungu, Rurumi, Thiba, Karima, Kiratina and Kasarani. Using simple random sampling, four villages were selected at Thiba location: Gakungu, Kasarani, Kiratina and Rurumi.

Probability proportional to size sampling technique was applied to determine the minimum number of households to be sampled per village. This sampling technique was adopted from another study that was carried out in the same area (Gichuki et al., 2019). Village chairpersons provided a list of all the households within the village. Using systematic sampling, the study selected households within each village. This was used since all villages have the same ecological features and schistosomiasis is a focal disease at the village. The first household was selected randomly at the center of the village. This was followed by selection of every fourth household in each direction from the first household surveyed. Consent was also sought prior to the commencement of the survey. To achieve consent, sensitization meetings were organized across the villages, during which community leaders and local administrators were informed about the study. The study recruited participants’ age ranged between 2 and 100 years.

### 2.4 Sample collection

The participants were provided with 2 screw-top containers (for holding stool and urine), a pair of gloves, disposable spatula, and clean newspaper in the morning. The screw-top containers were labeled with the date of collection and unique identification codes. All participants were taken through the steps for the collection of stool and urine. For instance, the participants were instructed on collecting the stool using a clean newspaper and placing it in the screw-top container before washing their hands. As for the urine, the participants were instructed to start peeing and then collect the “mid-stream” urine. The samples were handed in on the same day and taken to Kimbimbi Sub-county Hospital Laboratory for analysis. At the laboratory, some stool samples were used for Kato-Katz test, and about 100 mg of stool was stored in 2 ml tubes and preserved in absolute ethanol at 1:2 mass to volume ratio. Urine samples of 1 ml were similarly divided and placed in 2-ml tubes for preservation. The stool and urine samples in the 2 ml tubes were then stored at −20 °C before being transported to KEMRI laboratories in cooler boxes containing ice packs, for the qPCR and POC-CCA assay.

### 2.5 Microscopic examination

At the Kimbimbi Sub-County Hospital Laboratory, the stool samples were processed using the Kato-Katz test protocol and examined under a microscope to detect the eggs of *S. mansoni*. Each stool sample was prepared in a duplicate smear of 41.7 mg (Katz et al., 1972). The 2 thick smears prepared from the same stool were independently examined for *S. mansoni* eggs by two experienced laboratory technicians. Where the results showed discrepancy among slide readers (technicians), a senior experienced technician read the slide to break the tie. A random sample of 10% of all the negative and positive slides was reexamined by a third experienced laboratory technician for quality assurance.

### 2.6 POC-CCA assays

The POC-CCA assay was conducted at a temperature range of 24–30 °C as recommended by the manufacturer (Rapid Medical Diagnostics, Pretoria, South Africa). The results were read in a blinded manner by two technicians to guarantee quality. On the one hand, pink color on the test band and the standard band provided by the manufacturer indicated that *S. mansoni* was present. On the other hand, no color change on the test band and pink color observed on the control band indicated that *S. mansoni* was absent. Additionally, in cases where the absence of the control line showing that the results were invalid, the test was repeated using a new CCA cassette.

### 2.7 qPCR assays

The study used the MP Bio Fast DNA Spin kit for soil (MP Biomedical, Santa Ana, CA, USA), to extract the DNA of each of the fecal matter sample. The fecal matter sample was first added to a lysing matrix followed by a bead beating process to break the parasitic worms’ eggs to release the DNA. Bead beating was then followed up with several steps of DNA purification to remove the contaminants. The extracted DNA was finally eluted in 100 μl buffer provided by the manufacturer. The DNA extract was then stored at 4 °C awaiting the qPCR testing within a few hours or at −20 °C for long time storage. For quality control an extraction blank sample (reagents only) was used as a negative control.

The amplification and detection were carried out using a set of primers/probes that are complementary to a 121-bp tandem repeat sequence of the strain of *S. mansoni* described by Hamburger et al. (1991). The primers/probe sequence were Sm FW (5′-AAT CCG ACC AAC CGT TCT ATG-3′), Sm RV (5′-GCC CAC GCT CTC GCA AAT AA-3′) and Sm probe (5'-/56-FAM/ATC GTT GTA/ZEN/TCT CCG AAA CCA CTG GAC/3IABkFQ/-3′). The qPCR reaction mixture contained 0.7 μl of forward primer, 0.18 μl of reverse primer, 3.5 μl of TaqPath ProAmp Master Mix, 0.09 μl of probe, 0.53 μl of nuclease-free water and 2 μl of DNA. Each 96-well Fast MicroAmp qPCR plate (Applied Biosystems) was loaded with the thawed and vortexed DNA samples, and known negative and positive controls, all in duplicate (for quality control). The cycling parameters for the reaction included an initial incubation step at 50 °C for 2 min, followed by a denaturing step at 95 °C for 10 min, 40 cycles at 95 °C for 15 s, and 1 min at 61 °C for annealing and extension.

This reaction was run on a StepOnePlus™ Real-Time PCR System (Applied Biosystems), which provided exportable data illustrating the amplification threshold over all the 40 cycles. The results of each run were considered valid if the positive and the negative control worked. A DNA sample was considered negative if amplification was not observed from the duplicate wells (C_t_ = 0 or > 38). A DNA sample was considered positive when the threshold was attained within 38 cycles (C_t_ < 38).

### 2.8 Data management and analysis

Data were collected and entered into Excel sheets. Data management and analyses were performed using STATA version 15.1 (STATA Corporation, College Station, TX, USA). During the analysis, the following parameters were calculated: true positive (TP), true negative (TN), false positive (FP) and false negative (FN). TP is defined as the number of samples positive by all the three techniques (Kato-Katz, qPCR or POC-CCA); TN is the number of samples negative by all the three techniques; FP is the number of samples positive by qPCR or POC-CCA techniques but negative by Kato-Katz; FN is the number of samples negative by qPCR or POC-CCA techniques but positive by Kato-Katz. Accordingly, positive predictive values (PPV) were calculated as TP/(TP+FP) ​× ​100 (%) and negative predictive values (NPV) were calculated as TN/(TN ​+ ​FN) ​× ​100 (%). Further, sensitivity (Sn) was calculated as (TP)/(TP ​+ ​FN) ​× ​100 (%) and specificity (Sp) was calculated as TN/(TN ​+ ​FP) ​× ​100 (%).

Using Kato-Katz as the gold standard or ‘reference test’, the performance of qPCR and POC-CCA were evaluated by calculating the following performance measures at 95% confidence intervals (CIs): Sn, Sp, PPV, NPV, positive likelihood ratio (LR^+^), negative likelihood ratio (LR^−^) and Kappa score (Okoyo et al., 2018). Sensitivity and specificity were determined using a 2 × 2 contingency tables and their statistical significance compared using McNemar’s chi-square test (Riffenburgh, 2005). PPV and NPV were determined using weighted generalized score chi-square test for paired data (Kosinski, 2013). For each measure listed above, exact binomial with 95% CIs were calculated. Concordance (agreement) among the three diagnostic techniques was determined using the kappa statistics with 95% CIs and interpreted as per Landis and Koch (1977) classification. Additionally, the choice of the gold standard was varied to be either qPCR or the averaged results of all the three techniques, and accordingly, the performance measures of each of the technique was evaluated and compared. The infection prevalence of *S. mansoni* by each diagnostic technique was calculated at the village level and the 95% CIs estimated using binomial regression model.

## 3 Results

A total of 357 samples were collected from individuals aged between 2 and 100 years (mean age 28.8 years; standard deviation (SD) 17.7 years) in Gakungu (*n* = 87, 24.4%), Kasarani (*n* = 73, 20.5%), Kiratina (*n* = 104, 29.1%) and Rurumi (*n* = 93, 26%) villages in Mwea, Kirinyaga County. Samples were collected for 196 (54.9%) females and 161 (45.1%) males.

Comparative data for the prevalence of *S. mansoni* estimated across the four villages using the three diagnostic techniques, Kato-Katz, POC-CCA and qPCR, are provided in Table 1. The overall infection prevalence was 32.8% (95% CI: 28.2–38.0%), 62.5% (95% CI: 57.6–67.7%) and 72.8% (95% CI: 68.4–77.6%) using Kato-Katz, POC-CCA and qPCR techniques, respectively. Notably, the number of samples found to be positive by qPCR were higher compared to the other techniques. According to the village-level prevalence and averaging by all the three techniques, a higher number of infections was observed in Gakungu village (60.5%), followed by Kasarani (56.2%), Karatina (54.5%) and Rurumi (53.4%) villages. Similarly, on average, males had slightly higher prevalence compared to females, with prevalence by Kato-Katz being significantly different among the two genders (χ^2^ = 7.687, *P* = 0.006). According to the age groups, on average, higher number of infections were observed in adults (>14 years-old), followed by school-aged children (5–14 years-old), and least in pre-school-aged children (<5 years-old); however, the difference in prevalence between the three techniques was not significant (Table 1).

**Table 1:** Number of individuals examined and comparison of *Schistosoma mansoni* prevalence (%) using Kato-Katz, POC-CCA and qPCR among participants sampled in Mwea, Kirinyaga County.

Characteristic	No. examined (%)	Kato-Katz		POC-CCA		qPCR	
		No. positive (*n* = 117)	Prevalence (95% CI) (%)	No. positive (*n* = 223)	Prevalence (95% CI) (%)	No. positive (*n* = 260)	Prevalence (95% CI) (%)
Village							
Gakungu	87 (24.3)	35	40.2 (31.1–52.0)	59	67.8 (58.7–78.4)	64	73.6 (64.9–83.4)
Kasarani	73 (20.5)	20	27.4 (18.9–39.8)	45	61.6 (51.4–73.9)	58	79.5 (70.0–89.3)
Kiratina	104 (29.1)	33	31.7 (23.9–42.1)	64	61.5 (52.9–71.6)	73	70.2 (61.9–79.6)
Rurumi	93 (26.1)	29	31.2 (23.1–42.2)	55	59.1 (49.9–70.0)	65	69.9 (61.2–79.9)
Difference (χ^2^, *P*-value)	–	–	χ^2^ = 3.311, *P* = 0.346	–	χ^2^ = 1.560, *P* = 0.668	–	χ^2^ = 2.413, *P* = 0.491
Gender							
Female	196 (54.9)	52	26.5 (21.0–33.5)	118	60.2 (53.7–67.5)	143	73.0 (67.0–79.5)
Male	161 (45.1)	65	40.4 (33.5–48.7)	105	65.2 (58.3–73.0)	117	72.7 (66.1–79.9)
Difference (χ^2^, *P*-value)	–	–	χ^2^ = 7.687, *P* = 0.006^a^	–	χ^2^ = 0.948, *P* = 0.330	–	χ^2^ = 0.004, *P* = 0.951
Age group (years)							
< 5	14 (3.9)	1	7.1 (1.1–47.2)	10	71.4 (51.3–99.5)	9	64.3 (43.5–95.0)
5–14	86 (24.2)	28	32.6 (24.0–44.1)	46	53.5 (43.9–65.1)	62	72.1 (63.2–82.2)
> 14	256 (71.9)	87	34.0 (28.7–40.3)	166	64.8 (59.3–71.0)	188	73.4 (68.2–79.1)
Difference (χ^2^, *P*-value)	–	–	χ^2^ = 4.354, *P* = 0.113	–	χ^2^ = 4.047, *P* = 0.132	–	χ^2^ = 0.586, *P* = 0.746
Overall	357 (100)	117	32.8 (28.2–38.0)	223	62.5 (57.6–67.7)	260	72.8 (68.4–77.6)

*Note*: Statistical difference of the prevalence was assessed using Pearsonʼs chi-square test statistic.

a Statistically significant difference in prevalence.

Table 2 provides the true positives, true negatives and discrepancies for POC-CCA and qPCR using Kato-Katz as the gold standard. From the 357 samples examined, true positives were 25 (7.0%) and 114 (31.9%) for POC-CCA and qPCR, respectively, when compared with Kato-Katz as the gold standard. Accordingly, true negatives were 109 (30.5%) and 94 (26.3%), respectively, for the two techniques when compared to Kato-Katz. Further, 156 discrepancies (131 false positives and 25 false negatives) were recorded between POC-CCA and Kato-Katz, while 149 discrepancies (146 false positives and 3 false negatives) were recorded between qPCR and Kato-Katz.

**Table 2:** True positives, true negatives and discrepancies for POC-CCA and qPCR using Kato-Katz as the gold standard.

Diagnostic technique	Kato-Katz		
	Negative	Positive	Total
POC-CCA			
Negative	109	25	134
Positive	131	92	223
Total	240	117	357
qPCR			
Negative	94	3	97
Positive	146	114	260
Total	240	117	357

Similarly, Table 3 provides the true positives, true negatives and discrepancies for POC-CCA and Kato-Katz when using qPCR as the gold standard. From the 357 samples examined, true positives were 203 (56.9%) and 114 (31.9%) for POC-CCA and Kato-Katz, respectively, when compared with qPCR as the gold standard. Accordingly, true negatives were 77 (21.6%) and 94 (26.3%), respectively, for the two techniques when compared to qPCR. Further, 77 discrepancies (20 false positives and 57 false negatives) were recorded between POC-CCA and PCR, while 149 discrepancies (3 false positives and 146 false negatives) were recorded between Kato-Katz and qPCR.

**Table 3:** True positives, true negatives and discrepancies for POC-CCA and Kato-Katz using qPCR as the gold standard.

Diagnostic technique	qPCR		
	Negative	Positive	Total
POC-CCA			
Negative	77	57	134
Positive	20	203	223
Total	97	260	357
Kato-Katz			
Negative	94	146	240
Positive	3	114	117
Total	97	260	357

The performance measures (sensitivity, specificity, LR^+^, LR^−^, PPV, NPV) and the agreement for each of the techniques were evaluated under various scenarios: (i) using Kato-Katz as the gold standard; (ii) using qPCR as the gold standard; and (iii) using the averaged results from the three techniques as the gold standard.

Using Kato-Katz as the gold standard, POC-CCA sensitivity was 78.6% (95% CI: 70.1–85.7%) and specificity was 45.4% (95% CI: 39.0–51.9%). Overall, there was a slight/poor agreement between POC-CCA and Kato-Katz techniques in detecting *S. mansoni* infections (*κ* = 0.20, *P* < 0.001, concordance 56.3%; Table 4). On the other hand, qPCR sensitivity was 97.4% (95% CI: 92.7–99.5%) and specificity was 39.2% (95% CI: 33.0–45.7%), with an overall fair agreement between qPCR and Kato-Katz techniques in detecting *S. mansoni* infections (*κ* = 0.28, *P* < 0.001, concordance 58.3% (Table 4). From these results, the sensitivity of qPCR was significantly higher than that of POC-CCA (McNemar’s chi-square test, $\chi_{m}^{2}$ = 18.6, *P* < 0.001), while the specificity was the other way round ($\chi_{m}^{2}$ = 4.4, *P* = 0.036).

**Table 4:** Performance measures of POC-CCA and qPCR when Kato-Katz was used as the gold standard.

Diagnostic technique	Sensitivity (95% CI) (%)	Specificity (95% CI) (%)	LR^+^ (95% CI) (%)	LR^−^(95% CI) (%)	PPV (95% CI) (%)	NPV (95% CI) (%)	Kappa index (% Agreement)
POC-CCA	78.6 (70.1–85.7)	45.4 (39.0–51.9)	1.44 (1.24–1.67)	0.47 (0.32–0.68)	41.3 (34.7–48.0)	81.3 (73.7–87.5)	0.20 (56.3)
qPCR	97.4 (92.7–99.5)	39.2 (33.0–45.7)	1.60 (1.44–1.78)	0.07 (0.02–0.20)	43.8 (37.7–50.1)	96.9 (91.2–99.4)	0.28 (58.3)
Difference ($\chi_{m}^{2}$, *P*-value)	$\chi_{m}^{2}\;$ = 18.6, *P* < 0.001^a^	$\chi_{m}^{2}\;$ = 4.4, *P* = 0.036^a^	–	–	–	–	–

*Note*: Statistical difference of the sensitivity and specificity was obtained using McNemar’s chi-square test statistic.

*Abbreviations*: LR^+^, positive likelihood ratio; LR^−^, negative likelihood ratio; PPV, positive predictive value; NPV, negative predictive value.

a Statistically significant difference in sensitivity or specificity.

Using qPCR as the gold standard, Kato-Katz sensitivity was 43.8% (95% CI: 37.7–50.1%) and specificity was 96.9% (95% CI: 91.2–99.4%). Overall, there was a fair agreement between Kato-Katz and qPCR techniques in detecting *S. mansoni* infections (*κ* = 0.28, *P* < 0.001, concordance 58.3%; Table 5). On the other hand, POC-CCA sensitivity was 78.1% (95% CI: 72.5–83.0%) and specificity was 79.4% (95% CI: 70.0–86.9%), with an overall moderate agreement between POC-CCA and qPCR techniques in detecting *S. mansoni* infections (*κ* = 0.51, *P* < 0.001, concordance 78.4%; Table 5). From these results, it was observed that the sensitivity of Kato-Katz was significantly lower than that of POC-CCA ($\chi_{m}^{2}$ = 57.8, *P* < 0.001), while the specificity was significantly higher ($\chi_{m}^{2}$ = 15.2, *P* = 0.001).

**Table 5:** Performance measures of Kato-Katz and POC-CCA when qPCR was used as the gold standard.

Diagnostic technique	Sensitivity (95% CI) (%)	Specificity (95% CI) (%)	LR^+^ (95% CI) (%)	LR^−^(95% CI) (%)	PPV (95% CI) (%)	NPV (95% CI) (%)	Kappa index (% Agreement)
Kato-Katz	43.8 (37.7–50.1)	96.9 (91.2–99.4)	14.18 (4.61–43.56)	0.58 (0.52–0.65)	97.4 (92.7–99.5)	39.2 (33.0–45.7)	0.28 (58.3)
POC-CCA	78.1 (72.5–83.0)	79.4 (70.0–86.9)	3.79 (2.55–5.63)	0.28 (0.21–0.35)	91.0 (86.5–94.4)	57.5 (48.6–66.0)	0.51 (78.4)
Difference ($\chi_{m}^{2}$, *P*-value)	$\chi_{m}^{2}\;$ = 57.8, *P* < 0.001^a^	$\chi_{m}^{2}\;$ = 15.2, *P* = 0.001^a^	–	–	–	–	–

*Note*: Statistical difference of the sensitivity and specificity was obtained using McNemar’s chi-square test statistic.

*Abbreviations*: LR^+^, positive likelihood ratio; LR^−^, negative likelihood ratio; PPV, positive predictive value; NPV, negative predictive value.

a Statistically significant difference in sensitivity or specificity.

Finally, using the averaged results from the three techniques as the gold standard, Kato-Katz sensitivity was 41.6% (95% CI: 35.8–47.6%) and specificity was 100% (95% CI: 95.3–100%), with a fair agreement between Kato-Katz and the averaged results (*κ* = 0.23, *P* < 0.001, concordance 54.1%). POC-CCA sensitivity was 79.4% (95% CI: 74.2–83.9%) and specificity was 100% (95% CI: 95.3–100%), with an overall substantial agreement between POC-CCA and the averaged results (*κ* = 0.62, *P* < 0.001, concordance 83.8%). Further, qPCR sensitivity was 92.5% (95% CI: 88.8–95.3%) and specificity was 100% (95% CI: 95.3–100%), with an overall almost perfect agreement between qPCR and the averaged results (*κ* = 0.84, *P* < 0.001, concordance 94.1%; Table 6). From these results, it was observed that the sensitivity of three techniques varied considerably with the qPCR showing the highest sensitivity followed by POC-CCA and finally Kato-Katz with the lowest sensitivity. However, the specificity of the three techniques was satisfactory.

**Table 6:** Performance measures of Kato-Katz, POC-CCA and qPCR when the averaged results from the three techniques were used as the gold standard.

Diagnostic technique	Sensitivity (95% CI) (%)	Specificity (95% CI) (%)	LR^+^ (95% CI) (%)	LR^−^(95% CI) (%)	PPV (95% CI) (%)	NPV (95% CI) (%)	Kappa index (% Agreement)
Kato-Katz	41.6 (35.8–47.6)	100 (95.3–100)	–	0.58 (0.53–0.64)	100 (96.9–100)	31.7 (25.8–38.0)	0.23 (54.1)
POC-CCA	79.4 (74.2–83.9)	100 (95.3–100)	–	0.21 (0.16–0.26)	100 (98.4–100)	56.7 (47.9–65.2)	0.62 (83.8)
qPCR	92.5 (88.8–95.3)	100 (95.3–100)	–	0.07 (0.05–0.11)	100 (98.6–100)	78.4 (68.8–86.1)	0.84 (94.1)

*Abbreviations*: LR^+^, positive likelihood ratio; LR^−^, negative likelihood ratio; PPV, positive predictive value; NPV, negative predictive value.

## 4 Discussion

Health care practitioners need a rapid, easy to perform, sensitive, and specific point-of-care schistosomiasis diagnostic test (WHO, 2006). Precise diagnosis of schistosomiasis plays a fundamental role in the success of intervention programmes aimed at reducing the infection prevalence. This study was designed to evaluate and compare the performance of POC-CCA, qPCR and Kato-Katz techniques to diagnose *S. mansoni* infections. The results demonstrated that Kato-Katz was less effective and had low sensitivity compared to POC-CCA and qPCR, despite it being the preferred diagnostic technique because of it being easy to use in the field and relatively cheap. Further, this study showed that qPCR had superior sensitivity, followed by POC-CCA, and can be used as an alternative to Kato-Katz or to confirm the results obtained by Kato-Katz. Further, this analysis demonstrated that when a combined gold standard was used as the reference method, the sensitivity of the Kato-Katz technique was the lowest compared to the POC-CCA and qPCR.

Several studies have reported a higher sensitivity of the POC-CCA assay in detecting *S. mansoni* in endemic areas. Coulibaly et al. (2013) observed that a single POC-CCA assay is more sensitive than the Kato-Katz technique for diagnosis of schistosomiasis before and after administration of praziquantel. A study carried out in western Kenya also found that the CCA method has superior sensitivity than the Kato-Katz technique (Shane et al., 2011). Another study showed that using latent class analysis (LCA), the POC-CAA had a higher sensitivity than the Kato-Katz technique (Colley et al., 2017). These authors also observed, using multivariate modeling, that the POC-CCA was once again more sensitive than the Kato-Katz method. However, some authors argue that some factors, including the use of diuretics, hematuria, and urinary tract infections, may change the positivity of the POC-CCA, especially in weak positives (Lambertucci et al., 2017). This finding is consistent with Greter et al. (2016) who argued that a false-positive POC-CCA test is related to pregnancy, hematuria, and urinary tract infection. In other words, there are high chances for the POC-CCA assay to overestimate the prevalence of schistosomiasis.

Higher PPV and lower NPV when the Kato-Katz technique is used as the reference method indicates that the Kato-Katz method generally produces low numbers of false positives compared to POC-CCA. Previous studies support these results. For instance Pontes et al. (2003), found that the prevalence of schistosome infection in samples examined using the Kato-Katz technique (30.9%) was relatively lower than that of the samples examined by the PCR technique (38.1%). Another study performed in Kenya observed that the prevalence of *S. mansoni* infection analyzed by the POC-CCA test pre- and post-treatment was 26.5% and 21.4%, while the Kato-Katz technique assessed the prevalence at 4.9% and 1.5%, respectively (Okoyo et al., 2018). Further, Al-Shehri et al. (2018) found that the overall prevalence of schistosomiasis examined by Kato-Katz smears was 44.1% whereas the prevalence of samples tested by CCA dipstick was 67.4% and of those tested by PCR was 75.1%. Lodh et al. (2013) argued that Kato-Katz method only examines about 50 mg of fecal matter which is a small portion and thus, there is a high chance of missing some positive cases at low-level infections. The presence of false-positive and false-negative cases is an important matter that needs attention.

Regarding the distribution of the *S. mansoni*-positive and *S. mansoni*-negative cases of all the methods by age, it is clear that the group with most *S. mansoni*-positive cases was adults above 14 years. This could be because individuals above 14 years are the most active age group when it comes to farming in the Mwea irrigation scheme, therefore, this age group is relatively most exposed. This contrasts with previous studies which show age patterns that are not clear-cut when it comes to the intensity of schistosome infection (Verani et al., 2011; Coulibaly et al., 2013).

Another important observation is that there were 114 positive samples identified by both Kato-Katz and qPCR techniques, yet only three samples were identified to be negative by qPCR but positive by the Kato-Katz technique. However, a previous study has similarly reported false schistosomiasis-negative samples by the qPCR method. Allam et al. (2009) noted that the qPCR method showed negative results amongst samples considered to be positive. False-negative results using the qPCR method may be due to the degradation of the DNA of *S. mansoni* because of poor storage or poor transportation or presence of inhibitors that may have inhibited the amplification stage (Pontes et al., 2003).

Finally, in this study using the combined gold standard as the reference method, all the three techniques (qPCR, Kato-Katz and POC-CCA) had 100% specificity. This is because the presence of *S. mansoni*-specific DNA, *S. mansoni* antigen in urine or *S. mansoni* eggs in the stool, is highly specific. These findings are consistent with the study by Fuss et al. (2018) who indicated that the qPCR and Kato-Katz showed the highest specificity using LCA as the reference method to calculate the sensitivity and specificity.

## 5 Conclusions

The present study compared the performance measures of three different diagnostic techniques commonly used by schistosomiasis control programmes to diagnose *S. mansoni* infections under different transmission settings. The results demonstrated that Kato-Katz was less effective and had low sensitivity compared to POC-CCA and qPCR, despite it being the preferred diagnostic technique because of it being easy to use in the field and relatively cheap. Further, the study showed that qPCR had superior sensitivity, followed by POC-CCA, and can be used as alternatives to Kato-Katz or to confirm the results obtained by Kato-Katz. The disadvantages of the PCR technique is that it requires skilled personnel and specialized laboratory equipment, and the reagents are quite expensive (Obeng et al., 2008; Meurs et al., 2015). This diagnostic test can be advanced effectively through proper automation which will relatively reduce cost and consequently can be used for mass testing. The qPCR method can thus be an effective diagnostic test that is specific, sensitive and less laborious, and thus a reliable *S. mansoni* diagnostic test. This study also showed that there is no significant difference between different age groups.

## Funding

This study was funded by a KEMRI Internal Research Grant (IRG) ref no: KEMRI/IRG/041 for year 2017/2018. The funders had no role in the study design, data collection and analysis, decision to publish or preparation of the manuscript.

## Ethical approval

The study received ethical approval from the Kenya Medical Research Institute - Scientific and Ethics Review Unit (SERU No. 3672). The County Health Ministry, sub-county administration, and village elders granted permission to conduct the study. Prior to the survey, stakeholders’ meetings were held at the county and sub-county levels, after which sensitization meetings were held with chief and assistant chiefs from all locations and sub-locations. Village meetings were held at the villages led by the village elders. Written informed consent was obtained from all participants before they were enrolled in the study. Regarding children, assent was obtained after their parents or legal guardians provided consent all information and consent procedures were conducted in Kiswahili and/or Kikuyu (local languages). Finally, all study participants who turned positive for *S. mansoni* were treated with praziquantel (40 mg/kg), and those who turned positive for STHs were treated with albendazole (400 mg) after the study, according to the WHO guidelines (WHO, 2005).

## Declaration of competing interests

The authors declare that they have no known competing financial interests or personal relationships that could have appeared to influence the work reported in this paper.

## CRediT author statement

BC participated in the study design, data collection, laboratory procedures, analysis, and developed the draft of the manuscript. ES, CM and PG participated in study design and sample collection. CO and SK participated in analysis and revised the draft manuscript. SMN and DM participated in the study design and provided overall scientific guidance. All authors participated in the interpretation of the findings, and read and approved the final manuscript.

## References

[bib1] Al-Shehri H., Koukounari A., Stanton M.C., Adriko M., Arinaitwe M., Atuhaire A. (2018). Surveillance of intestinal schistosomiasis during control: a comparison of four diagnostic tests across five Ugandan primary schools in the Lake Albert region. Parasitology.

[bib2] Allam A.F., Kader O., Zaki A., Youssef Shehab A., Farag H.F. (2009). Assessing the marginal error in diagnosis and cure of *Schistosoma mansoni* in areas of low endemicity using Percoll and PCR techniques. Trop. Med. Int. Health.

[bib3] Ashton R.A., Stewart B.T., Petty N., Lado M., Finn T., Brooker S., Kolaczinski J.H. (2011). Accuracy of circulating cathodic antigen tests for rapid mapping of *Schistosoma mansoni* and *S. haematobium* infections in southern Sudan. Trop. Med. Int. Health.

[bib4] Brooker S., Hotez P.J., Bundy D.A.P. (2010). The global atlas of helminth infection: mapping the way forward in neglected tropical disease control. PLoS Negl. Trop. Dis..

[bib5] Carvalho Do Espiŕito-Santo M.C., Pinto P.L., Gargioni C., Alvarado-Mora M.V., Pagliusi Castilho V.L. (2014). Detection of *Schistosoma mansoni* antibodies in a low-endemicity area using indirect immunofluorescence and circumoval precipitin test. Am. J. Trop. Med. Hyg..

[bib6] Chernet A., Kling K., Sydow V., Kuenzli E., Hatz C., Utzinger J. (2017). Accuracy of diagnostic tests for *Schistosoma mansoni* infection in asymptomatic Eritrean refugees: serology and point-of-care circulating cathodic antigen against stool microscopy. Clin. Infect. Dis..

[bib7] Clark N.J., Umulisa I., Ruberanziza E., Owada K., Colley D.G., Ortu G. (2019). Mapping *Schistosoma mansoni* endemicity in Rwanda: a critical assessment of geographical disparities arising from circulating cathodic antigen versus Kato-Katz diagnostics. PLoS Negl. Trop. Dis..

[bib8] Colley D.G., Andros T.S., Campbell C.H. (2017). Schistosomiasis is more prevalent than previously thought: what does it mean for public health goals, policies, strategies, guidelines and intervention programs?. Infect. Dis. Poverty.

[bib9] Coulibaly J.T., N’gbesso Y.K., N’guessan N.A., Winkler M.S., Utzinger J., N’goran E.K. (2013). Epidemiology of schistosomiasis in two high-risk communities of south Côte dʼIvoire with particular emphasis on pre-school-aged children. Am. J. Trop. Med. Hyg..

[bib10] Deol A.K., Fleming F.M., Calvo-Urbano B., Walker M., Bucumi V., Gnandou I. (2019). Schistosomiasis - assessing progress toward the 2020 and 2025 global goals. N. Engl. J. Med..

[bib11] Fisher A.C. (1934). A study of the schistosomiasis of the Stanleyville district of the Belgian Congo. Trans. R. Soc. Trop. Med. Hyg..

[bib12] Fuss A., Mazigo H.D., Tappe D., Kasang C., Mueller A. (2018). Comparison of sensitivity and specificity of three diagnostic tests to detect *Schistosoma mansoni* infections in school children in Mwanza region, Tanzania. PloS One.

[bib13] Gichuki P.M., Mbugua G., Kiplelgo E.K., Irungu T.W., Mwandawiro C. (2019). Long term school based deworming against soil-transmitted helminths also benefits the untreated adult population: results from a community-wide cross-sectional survey. J. Trop. Med..

[bib14] Glinz D., Silué K.D., Knopp S., Lohourignon L.K., Yao K.P., Steinmann P. (2010). Comparing diagnostic accuracy of Kato-Katz, Koga agar plate, ether-concentration, and FLOTAC for *Schistosoma mansoni* and soil-transmitted helminths. PLoS Negl. Trop. Dis..

[bib15] Greter H., Krauth S.J., Ngandolo B.N.R., Alfaroukh I.O., Zinsstag J., Utzinger J. (2016). Validation of a point-of-care circulating cathodic antigen urine cassette test for *Schistosoma mansoni* diagnosis in the Sahel, and potential cross-reaction in pregnancy. Am. J. Trop. Med. Hyg..

[bib16] Haggag S.H., Abdullah S.M. (2011). Molecular diagnosis of *Schistosoma mansoni* infection in human serum and feces by using polymerase chain reaction. Life Sci. J..

[bib17] Hamburger J., Turetski T., Kapeller I., Deresiewicz R. (1991). Highly repeated short DNA sequences in the genome of *Schistosoma mansoni* recognized by a species-specific probe. Mol. Biochem. Parasitol..

[bib18] Hotez P.J., Alvarado M., Basáñez M.G., Bolliger I., Bourne R., Boussinesq M. (2014). The global burden of disease study 2010: interpretation and implications for the neglected tropical diseases. PLoS Negl. Trop. Dis..

[bib19] Katz N., Chaves A., Pellegrino J. (1972). A simple device for quantitative stool thick-smear technique in schistosomiasis mansoni. Rev. Inst. Med. Trop. Sao Paulo.

[bib20] Kildemoes A.O., Vennervald B.J., Tukahebwa E.M., Kabatereine N.B., Magnussen P., de Dood C.J. (2017). Rapid clearance of *Schistosoma mansoni* circulating cathodic antigen after treatment shown by urine strip tests in a Ugandan fishing community - relevance for monitoring treatment efficacy and re-infection. PLoS Negl. Trop. Dis..

[bib21] Kittur N., Castleman J.D., Campbell C.H., King C.H., Colley D.G. (2016). Comparison of *Schistosoma mansoni* prevalence and intensity of infection, as determined by the circulating cathodic antigen urine assay or by the kato-katz fecal assay: a systematic review. Am. J. Trop. Med. Hyg..

[bib22] KNBS (2010). https://s3-eu-west-1.amazonaws.com/s3.sourceafrica.net/documents/21195/Census-2009.pdf.

[bib23] Kosinski A.S. (2013). A weighted generalized score statistic for comparison of predictive values of diagnostic tests. Stat. Med..

[bib35] Lambertucci J.R., Teixeira Ferreira F., Fidelis T.A., Pereira T.A., Otoni A., Campos Queiroz L. (2017). Sensitivity and specificity of the circulating cathodic antigen rapid urine test in the diagnosis of schistosomiasis mansoni infection and evaluation of morbidity in a low-endemic area in Brazil. Rev. Soc. Bras. Med. Trop..

[bib24] Landis J., Koch G. (1977). The measurement of observer agreement for categorical data. Biometrics.

[bib25] Lodh N., Mwansa J.C.L., Mutengo M.M., Shiff C.J. (2013). Diagnosis of *Schistosoma mansoni* without the stool: comparison of three diagnostic tests to detect *Schiostosoma mansoni* infection from filtered urine in Zambia. Am. J. Trop. Med. Hyg..

[bib26] Masaku J., Madigu N., Okoyo C., Njenga S.M. (2015). Current status of *Schistosoma mansoni* and the factors associated with infection two years following mass drug administration programme among primary school children in Mwea irrigation scheme: a cross-sectional study. BMC Publ. Health.

[bib27] Meurs L., Brienen E., Mbow M., Ochola E.A., Mboup S., Karanj D.M.S. (2015). Is PCR the next reference standard for the diagnosis of *Schistosoma* in stool? A comparison with microscopy in Senegal and Kenya. PLoS Negl. Trop. Dis..

[bib28] Noya O., Alarcón De Noya B., Losada S., Colmenares C., Guzmán C., Lorenzo M.A., Bermúdez H. (2002). Laboratory diagnosis of schistosomiasis in areas of low transmission. A review of a line of research. Mem. Inst. Oswaldo Cruz.

[bib29] Obeng B.B., Aryeetey Y.A., De Dood C.J., Amoah A.S., Larbi I.A., Deelder A.M. (2008). Application of a circulating-cathodic-antigen (CCA) strip test and real-time PCR, in comparison with microscopy, for the detection of *Schistosoma haematobium* in urine samples from Ghana. Ann. Trop. Med. Parasitol..

[bib30] Ochodo E.A., Gopalakrishna G., Spek B., Reitsma J.B., van Lieshout L., Polman K. (2015). Circulating antigen tests and urine reagent strips for diagnosis of active schistosomiasis in endemic areas. Cochrane Database Syst. Rev..

[bib31] Okoyo C., Nikolay B., Kihara J., Simiyu E., Garn J.V., Freeman M.C. (2016). Monitoring the impact of a national school based deworming programme on soil-transmitted helminths in Kenya: the first three years, 2012–2014. Parasit. Vectors.

[bib32] Okoyo C., Simiyu E., Njenga S.M., Mwandawiro C. (2018). Comparing the performance of circulating cathodic antigen and Kato-Katz techniques in evaluating *Schistosoma mansoni* infection in areas with low prevalence in selected counties of Kenya: a cross-sectional study. BMC Publ. Health.

[bib33] Pontes L.A., Oliveira M.C., Katz N., Dias-Neto E., Rabello A. (2003). Comparison of a polymerase chain reaction and the Kato-Katz technique for diagnosing infection with *Schistosoma mansoni*. Am. J. Trop. Med. Hyg..

[bib34] Riffenburgh R.H. (2005). Statistics in Medicine.

[bib36] Shane H.L., Verani J.R., Abudho B., Montgomery S.P., Blackstock A.J., Mwinzi P.N.M. (2011). Evaluation of urine CCA assays for detection of *Schistosoma mansoni* infection in Western Kenya. PLoS Negl. Trop. Dis..

[bib37] Song J., Liu C., Bais S., Mauk M.G., Bau H.H., Greenberg R.M. (2015). Molecular detection of schistosome infections with a disposable microfluidic cassette. PLoS Negl. Trop. Dis..

[bib38] Sousa-Figueiredo J.C., Basáñez M.-G., Khamis I.S., Garba A., Rollinson D., Stothard J.R. (2009). Measuring morbidity associated with urinary schistosomiasis: assessing levels of excreted urine albumin and urinary tract pathologies. PLoS Negl. Trop. Dis..

[bib39] Steinmann P., Keiser J., Bos R., Tanner M., Utzinger J. (2006). Schistosomiasis and water resources development: systematic review, meta-analysis, and estimates of people at risk. Lancet Infect. Dis..

[bib40] Taman A., El-Beshbishi S. (2019). Laboratory diagnosis of schistosomiasis mansoni: current status and future trends. Asian Pac. J. Trop. Med..

[bib41] Tchuem Tchuenté L.A. (2011). Control of soil-transmitted helminths in sub-Saharan Africa: diagnosis, drug efficacy concerns and challenges. Acta Trop..

[bib42] Truscott J.E., Déirdre Hollingsworth T., Brooker S.J., Anderson R.M. (2014). Can chemotherapy alone eliminate the transmission of soil transmitted helminths?. Parasit. Vectors.

[bib43] Utzinger J., Becker S.L., van Lieshout L., van Dam G.J., Knopp S. (2015). New diagnostic tools in schistosomiasis. Clin. Microbiol. Infect..

[bib44] Verani J.R., Abudho B., Montgomery S.P., Mwinzi P.N.M., Shane H.L., Butler S.E. (2011). Schistosomiasis among young children in Usoma, Kenya. Am. J. Trop. Med. Hyg..

[bib45] WHO (2006).

[bib46] WHO (2011).

[bib47] WHO (2012).

[bib48] WHO (2020). https://www.who.int/news-room/fact-sheets/detail/schistosomiasis.

